# Improvements in Hypodermic Injection

**Published:** 1882-03

**Authors:** 


### IMPROVEMENTS IN HYPODERMIC INJECTION.

Dr. Mason recommends the following as the best way of dealing with the piston of the hypodermic syringe when its packing gets worn and loose so that it does not work readily. Remove the small nut at the end of the piston and take half of the packing off (it is usually in two parts) and place between them a piece of chamois-skin. Cut it round leaving it somewhat larger than the packing. It will absorb water, swell, and completely fill the barrel. A trial of this will convince the most skeptical of its value over all other devices to do away with the most annoying feature connected with the use of the syringe.—*Med. Times and Gaz.*

